# A Pretty Kettle of Fish: A Review on the Current Challenges in Mediterranean Teleost Reproduction

**DOI:** 10.3390/ani14111597

**Published:** 2024-05-28

**Authors:** Marta Lombó, Christian Giommi, Matteo Zarantoniello, Giulia Chemello

**Affiliations:** 1 Department of Life and Environmental Sciences (DiSVA), Università Politecnica delle Marche, 60131 Ancona, Italy; mloma@unileon.es (M.L.); c.giommi@pm.univpm.it (C.G.); 2 INBB—Consorzio Interuniversitario di Biosistemi e Biostrutture, 00136 Roma, Italy; 3 Department of Molecular Biology, Faculty of Biology and Environmental Sciences, Universidad de León, 24071 León, Spain

**Keywords:** reproductive performance, aquaculture, climate change, water pollution, fish diet, overfishing

## Abstract

**Simple Summary:**

In the Mediterranean region, teleost species are facing diverse challenges resulting from the combined effects of climate change, pollution, and overfishing. The survival of these species and the overall stability of the ecosystem are closely linked to their reproductive success and their capacity to produce healthy offspring. Teleost reproduction is a crucial aspect in aquaculture as well since it ensures the productivity of high-quality animal proteins as an alternative to overexploited wild stocks. Therefore, deep knowledge on the factors influencing the reproduction of species devoted to aquaculture at different levels is necessary to propose efficient management approaches, aiming to identify sustainable farming techniques. To attain this goal, it is initially necessary to comprehend the molecular and physiological mechanisms that regulate various levels of the reproductive process in a changing scenario. The use of model organisms allows scientists to test specific stressors without external interference, thanks to their availability, easier handling, and biological characteristics. However, caution is needed to avoid overestimating their applicability to other species. The present review aims to explore the current factors affecting reproduction in wild, farmed, and model teleosts.

**Abstract:**

The Mediterranean region is facing several environmental changes and pollution issues. Teleosts are particularly sensitive to these challenges due to their intricate reproductive biology and reliance on specific environmental cues for successful reproduction. Wild populations struggle with the triad of climate change, environmental contamination, and overfishing, which can deeply affect reproductive success and population dynamics. In farmed species, abiotic factors affecting reproduction are easier to control, whereas finding alternatives to conventional diets for farmed teleosts is crucial for enhancing broodstock health, reproductive success, and the sustainability of the aquaculture sector. Addressing these challenges involves ongoing research into formulating specialized diets, optimizing feeding strategies, and developing alternative and sustainable feed ingredients. To achieve a deeper comprehension of these challenges, studies employing model species have emerged as pivotal tools. These models offer advantages in understanding reproductive mechanisms due to their well-defined physiology, genetic tractability, and ease of manipulation. Yet, while providing invaluable insights, their applicability to diverse species remains constrained by inherent variations across taxa and oversimplification of complex environmental interactions, thus limiting the extrapolation of the scientific findings. Bridging these gaps necessitates multidisciplinary approaches, emphasizing conservation efforts for wild species and tailored nutritional strategies for aquaculture, thereby fostering sustainable teleost reproduction in the Mediterranean.

## 1. Introduction

The Mediterranean Sea is a marginal and semi-enclosed sea situated at the crossroads of Africa, Europe, and Asia. Since ancient times, it has been an important route for merchants and travelers and, currently, it represents an essential hub for global economy and trade [1]. The Mediterranean Sea is characterized by its semi-enclosed nature, limited connection to the Atlantic Ocean, and high variability in temperature and salinity. These environmental factors shape the unique ecosystems found within its waters [2]. As a result of that, it has long been considered a focal point of scientific inquiry and environmental concern [3,4]. Although it represents only 0.82% of the sea water surface, the Mediterranean Sea harbors nearly 17,000 known marine species, representing 4–18% of global marine biodiversity. Regarding fish, teleost species are the most abundant, representing 85% of Mediterranean fish [5]. These bony fish, characterized by their advanced reproductive systems, are vital components of the Mediterranean ecosystem, playing key roles in nutrient cycling, trophic interactions, and maintaining ecological balance [6]. In the last decades, the reproduction of teleosts has been threatened by the ongoing environmental changes and the increasing anthropogenic impacts [7].

### 1.1. Mediterranean Teleosts in a Changing Environment

Climate change can exert an influence on the marine environment through various processes, directly and indirectly altering the stability of environmental conditions. Rising sea temperatures and declining oceanic pH levels can induce ocean acidification, driven by the increased absorption of atmospheric carbon dioxide (CO_2_) by the oceans. Additionally, climate change has the potential to disturb ocean circulation patterns, resulting in alterations in currents and upwelling systems. Finally, climate fluctuations could induce hypoxia, shifts in precipitation patterns, and an increase in the frequency and intensity of extreme weather events, such as hurricanes and typhoons [8,9,10].

These variations can alter the reproductive success of teleosts by affecting the proper function of the brain–pituitary–gonadal axis (HPG) at different levels [11]. Temperature is one of the predominant factors influencing the reproductive processes of teleosts. Mediterranean surface waters are warming up at a rate > 2 °C/100 year along the whole water column [12]. The sensitivity of fish to thermal fluctuations varies, depending not only on the species and life stages but also on the range of thermal tolerance fish display [13,14]. Considering that most teleost species are poikilotherms, changes in temperature can impact different vital parameters, such as metabolic rate and growth, natural death, and reproductive success [7]. Another factor with outstanding contribution is salinity, which is often linked to climate-induced alterations in precipitation patterns. Slight shifts in salinity levels can affect the osmoregulatory mechanisms of teleosts, potentially leading to reduced hatching success and larval survival [15]. Altogether, these environmental stressors collectively contribute to the vulnerability of teleost reproductive success in the Mediterranean.

### 1.2. Impact of Anthropic Activities

The Mediterranean region is currently home to over 525 million people and this figure is expected to increase by an additional 130 million by 2050, particularly in the eastern and southern countries. Additionally, the Mediterranean countries remain the most important global tourist destination, welcoming over 360 million international tourists annually [16]. The concentration of population along the coastal trips has led to an increase in waste production, discharges of wastewater, and pressure on natural resources. Consequently, human activities exacerbate the challenges faced by teleosts in the Mediterranean. A major threat specific to the Mediterranean concerns fishery-related activities, with 88% of stocks being overfished [16]. The impacts of fisheries go beyond stocks; they affect seafloor habitats, including benthic fauna damage, and contribute to marine litter and microplastic generation [12]. Overfishing has led to a decline in teleost populations, affecting both their abundance and age structure. This can disrupt the intricate balance of reproductive dynamics, with potential consequences for the long-term sustainability of teleost populations [17].

The introduction of non-native species, whether intentionally or inadvertently, can also disrupt the reproductive ecology of native teleosts. Competition for resources, predation pressure, and disease transmission associated with invasive species can all interfere with successful teleost reproduction and survival [12]. In this regard, compared to the other regional seas, the Mediterranean has the highest number of invasive species that can jeopardize both the survival and reproduction of native species [16]. Moreover, pollutants, including heavy metals, (micro)plastics, agricultural runoffs, pharmaceuticals, and personal care products, have become pervasive in the Mediterranean. These contaminants can bioaccumulate in fish tissues [18] and can even be maternally transmitted to the offspring through the yolk, as demonstrated in model species [19]. The consequences range from impaired gametogenesis to skewed sex ratios, posing serious threats to the future of teleost populations in the region [20]. Nowadays, new approaches have been developed to monitor the levels of pollutants in sea water and in invertebrates and fish tissues. The derived results could provide useful information about the type and concentrations of different chemicals and will enable the correlation of fish survival and welfare with specific polluted conditions [21,22].

### 1.3. Implications for Teleost Farming

The aquaculture sector in the Mediterranean Sea represents the fastest-growing industry in food production, especially finfish farming [23]. Farmed teleosts help alleviate pressure on wild fish populations by offering an alternative source of seafood. However, as the challenges intensify for wild teleost populations, the aquaculture industry, which mostly relies on the breeding success of farmed species, faces its own set of obstacles [24]. Understanding how environmental changes and anthropogenic impacts influence the reproduction of teleosts is also crucial for developing sustainable aquaculture practices. In farmed species, it has been proven that adjustments in photoperiod, sea temperature, density, and salinity may ensure optimal reproductive conditions, as reviewed by Mylonas and Zohar [25]. Dietary factors can also influence reproductive performance and progeny survival of farmed species since the nutritional composition of feed provided in aquaculture settings may differ significantly from the natural diet of teleosts [26,27]. In fact, the dietary requirements of broodstock as well as those of larvae remain one of the critical points in aquaculture since they depend on the availability of nutrients and raw materials, and they can imbalance the ratio of inputs to outputs [28].

### 1.4. Model Species and Predictive Studies

To gain insights into the effects of environmental changes on teleost reproduction, researchers often turn to model species, such as zebrafish (*Danio rerio*) and medaka (*Oryzias latipes*). These species, with their rapid reproduction, transparency in early developmental stages, and genetic tractability, offer valuable tools for studying the impacts of temperature, salinity, and pollutants on teleost reproduction [29]. In addition, zebrafish can represent a useful model organism to conduct preliminary evaluations of innovative dietary formulations. In fact, due to their features, zebrafish can (i) simplify the evaluation of a wide range of alternative ingredients, tested at different inclusion levels, compared to the costly and more time-consuming feeding trials conducted on farmed fish, and (ii) enable a deeper understanding of the molecular mechanisms involved in nutrient metabolism [30,31]. Despite not being native to the Mediterranean, the findings from zebrafish and medaka studies can provide valuable insights into the potential challenges faced by native teleosts. It is noteworthy that studies on model species may oversimplify the complex interactions among different factors, neglecting the intricate ecological relationships that impact reproductive processes.

In this review, we have compiled information about the different factors affecting the reproductive performance of wild and farmed teleost species, focusing on those of the Mediterranean Sea. Moreover, we describe the most outstanding results obtained using model species to shed light on the impact of environmental pollution on teleost reproduction.

## 2. Reproductive Challenges in Wild Teleost Populations

In the present global context, the significant and irreversible alterations in marine conditions result from the combined impact of three primary stressors: climate change, environmental pollution, and overfishing [32]. The influence of these events on marine ecosystems depends on their close correlation. Indeed, climate change has the potential to alter both the toxicity and fate of marine contaminants as well as the extent of fishing im-pact [8,33,34]. The environmental distribution of persistent organic pollutants (POPs) is closely tied to sea temperature, organic carbon content, and their recycling among environmental reservoirs [34]. Alterations in environmental parameters affect the distribution, species composition, seasonality, and reproduction in both marine and freshwater eco-systems, thereby influencing the availability of fisheries’ resources. Consequently, to meet market demand, fishing activities may shift to other targets, intensifying efforts on new species or smaller specimens, often during their vulnerable life stages [33]. However, marine teleosts, which exhibit the most diverse reproductive strategies among vertebrates, may display varied responses when subjected to the same conditions. This complexity makes it challenging to generalize and predict the potential consequences of specific stressors. Therefore, research has focused on investigating aspects of the reproductive cycle shared by different species, regardless of their reproductive strategy, such as the development and growth of gametes and their quality and the preparation for the subsequent reproductive season (e.g., proliferation of germ cells in iteroparous species) [35].

### 2.1. Climate Change

Several reviews have detailed the impact of climate change on teleost reproductive physiology, focusing mainly on global warming, which has exhibited the most concerning effects [7,11,36,37,38]. For the record, in temperate teleosts, including those inhabiting the Mediterranean area, the photoperiod has been considered the primary factor influencing the onset of the reproductive cycle [39]. While the photoperiod itself is not directly affected by climate change, the migration of many marine species towards the poles in response to global warming has resulted in significant shifts in their photoperiodic reproductive periods [40]. This phenomenon may lead to anticipated delays and a shorter reproductive season [37].

In this review, we refrained from discussing the intricate molecular mechanisms governing reproduction impacted by variations in environmental temperature or other parameters. This decision has been made to avoid presenting redundant information already extensively covered in the afore-mentioned literature, although we briefly summarized the main effects observed in laboratory trials. The observed results highlighted that temperature fluctuations possess the capability to influence the HPG axis at multiple levels, thereby affecting hormone synthesis, action, and structure [11]. Departure from the optimal thermal range also exerts an impact on gamete development, spawning timing, and egg quality in female specimens [7]. Increasing temperature and hypoxia reduce sperm motility and increase the sperm oxygen consumption, affecting the quality of spermatozoa during fertilization [41]. Hypoxic events also have the potential to influence gonadal steroidogenesis, thereby affecting the availability of cholesterol precursors for steroid production or directly impacting aromatase activity, as reported by Servilli et al. [37]. Notable correlations were observed between changes in salinity or water acidification and a reduction in sperm quality and reproductive output [42,43].

### 2.2. Environmental Contaminations Move to Next Page

Numerous chemical substances released into the aquatic environment are categorized as persistent organic pollutants (POPs) due to their demonstrated high toxicity, capacity for long-range transport, persistence, and bioaccumulation potential [44]. Within this classification, certain pesticides like dichlorodiphenyltrichloroethane (DDT) are included, while other compounds, such as pharmaceutical products, display POP-like characteristics by persisting in aquatic environments and exhibiting the potential for environmental accumulation [44,45].

Some POPs (polychlorinated biphenyls (PCBs), dichlorodiphenyltrichloroethane (DDT)) express their toxicity by interfering with the endocrine pathway (synthesis, secretion, transport, or elimination) or interacting with receptors changing hormonal responses during fish reproduction and therefore have been named endocrine disruptors (EDCs) [46]. General mechanisms by which contaminants affect the normal reproductive process in fish are described in Section 4.

As a result of extensive anthropization and industrialization, Mediterranean coastal areas are major recipients of pollutants [47]. Indeed, the literature has documented the presence of various pesticides, pharmaceuticals products, and EDCs in sediments, seawater, and fish species within the Mediterranean regions [48,49,50,51,52,53,54]. The first study investigating the occurrence of various contaminants, in open surface water, concerned the western Mediterranean Sea [54]. From the 70 total samples collected in 10 different sampling areas, 3 pesticides and 11 pharmaceuticals and personal care products were detected. Other studies investigated the presence of various contaminants in fish from different areas of the Mediterranean Sea, as stated below.

Organochlorine pesticides including DDT and PCBs were detected in different teleost species from different areas of the Adriatic Sea [52,53]. Bisphenol A (BPA), 4-nonylphenol, and 4-t-octylphenol were found in red mullet (*Mullus barbatus*) from the Tyrrhenian Sea [48]. High levels of BPA were also found in the muscle of different fish species in the Cabrera Marine Protected Area (Balearic Islands) [49]. BPA and 4-alkylphenols were detected in sediments and seawater of two coastal areas of the Iberian Peninsula [50]. Different PCBs and organochlorines were detected in European anchovies from the Adriatic Sea [51].

Finally, the presence of heavy metals in the waters and fish species of the Mediterranean Sea has also been a matter of concern for a considerable period, and investigations into their presence persist to the present day, as reported in different recent publications [55,56,57].

### 2.3. Overfishing

Over the last twenty years in the Mediterranean area, different wild stocks have experienced a decline, while their exploitation levels have increased [58]. Generally, the overall impact of overfishing surpasses, to varying degrees, the direct impact of global warming on species and ecosystem diversity, as well as ecosystem functioning. This trend is likely to intensify in the coming decades [59]. Both the direct and indirect effects of fishing activity on the survival of a stock highly depend on the species’ reproductive strategy. Adult males and females and immature and mature specimens exhibit different behaviors during the reproductive season. These behavioral differences may lead to the selective removal of specific subgroups within the population, consequently altering the sex ratio and size distribution. This reduction in potential mate encounters can thereby decrease reproductive output [60]. Independently of the seasonal period, specific fishing techniques (such as bottom trawling and dredging) may alter the spawning habitat of certain demersal and benthic species. An interesting example of the aforementioned discussion is represented by the case study on the red mullet (*Mullus barbatus*) in the Gulf of Castellammare (northwestern Sicily, central Mediterranean). This study reported a significant increase in the spawning stock and recruitment pattern of red mullet after a 14-year ban on trawling in that area [61].

It is important to emphasize that the impacts of fishing activities on the reproduction of wild populations did not only concern large naval fleets; artisanal fisheries can also influence the survival of Mediterranean coastal species [62]. This issue is particularly significant, given that artisanal fisheries represent approximately 85% of the Mediterranean fishing fleet [63].

Among the consequences of fishing overexploitation, fluctuations in life history traits of marine fish, such as changes in the reproductive period and in size or age at first maturity, are the most common [64]. This phenomenon has been observed in species with long life cycles, such as the European hake (*Merluccius merluccius*) in the western Mediterranean Sea, although, in this study, the effects on the long-term dynamics of European hake population have been attributed to both fishing and hydroclimatic variability [65].

This example demonstrates how the impact of fishing on wild stocks depends on their interaction with other factors, including environmental conditions. Indeed, a species already stressed by adverse climate changes or contamination may not be able to thrive under exploitation. Conversely, fishing activity has the potential to disrupt the ability of a wild stock to endure or adapt to climate changes [8,63]. Given the critical nature of the current situation, immediate and effective management is essential to safeguard biodiversity and ensure the sustainability of fisheries’ resources for future generations.

### 2.4. Reproductive Challenges in Mediterranean Area

Due to the variable nature of all the forces influencing the intricate process of fish reproduction, it is evident that neither laboratory trials nor predictive models can comprehensively incorporate all factors impacting the reproductive cycle of wild teleost stocks. Our current level of knowledge is still inadequate for making precise predictions beyond general assumptions, except for the relatively few species that have garnered research interest. The main deficiency lies in the limited understanding of reproductive plasticity among teleost species when adapting over multiple generations, for instance in scenarios characterized by gradual temperature increases [37].

Though it is challenging to identify the predominant stressor driving changes in the reproductive cycle of a species, different populations have experienced alterations in specific reproductive traits in the Mediterranean area. These changes are attributed to variations in environmental conditions associated with climate change, contamination, and high exploitation over the years. An exemplary demonstration of population dynamics influenced by the combination of these three stressors is observed in the case of small pelagic species such as European sardines (*Sardina pilchardus*) and European anchovies (*Engraulis encrasicolus*) [66].

These two small pelagic species stand out as the most significant in terms of abundance, ecological relevance, and economic value, being the principal targets for purse seiners and pelagic trawlers in the Mediterranean Sea [67]. European sardines and European anchovies typically experience substantial fluctuations in their abundance and biomass due to their strong reliance on environmental variability [68]. As a result, a growing focus has been developed on exploring the correlation with ongoing environmental changes to effectively implement conservation programs for these species. The observed decline in the abundance of European anchovy eggs and larvae in the north-west Mediterranean Sea during surveys conducted in the 1980s, 2000s, and 2010s was attributed to the gradual increase in seawater temperature and salinity, paired with a concurrent decrease in chlorophyll a levels [69]. Basilone and collaborators (2021) during a 9-year-long time series obtained within the European Data Collection Framework (DCF; EC 665/2008) provided basic knowledge on the reproductive biology of European sardines in the central Mediterranean Sea [70].

Along the analyzed period, the size at first maturity ranged from 108 to 124 mm (total length) for females and from 102 to 122 mm for males, with significant differences among years linked to sardines’ flexibility to environmental variability [70].

An evident decline in stock population with possible consequences on the reproductive parameters was observed for sardines from the Adriatic population. Indeed, a general reduction in the mean size (15.5 cm the highest size class observed) and age (greater than 2 years old but less than 3 years) of the population was observed, together with a predominance of male specimens in the population [71]. These data differed from those obtained in previous surveys, where sardines’ total length was observed up to 17.0 cm and 21.0 cm [72,73,74]. The situation is likely to impact the reproductive success and overall stability of the species, particularly due to the reduction in female specimens. A higher number of females is considered advantageous for the population, as it correlates with a greater reproductive rate [75].

An alarming effect of endocrine disruptors was observed in the European anchovies from the western Adriatic Sea, showing the effects of overexposure to contaminants. In male specimens where PCBs and organochlorines were detected, transcription of vitellogenin, vitellogenin receptors, and genes encoding zona radiata proteins was observed in gonad and liver samples. Additionally, histological analysis identified intersex characteristics in 13% of the analysed testes [51].

A similar scenario was observed in two species of top predators inhabiting the Mediterranean Sea: bluefin tuna (*Thunnus thynnus*) and swordfish (*Xiphias gladius*), for which high induction of vitellogenin and zona radiata proteins was detected in the serum of male specimens of these species [76,77]. In *Xiphias gladius,* evidence of intersex in male gonads was also observed [78,79].

Although presently there are limited records of reproductive alterations in teleost that can be unequivocally linked to specific factors, the consequences of environmental changes resulting from climate change, contamination, and overfishing may manifest across various species in the years to come. Therefore, anticipating the future threats to the marine ecosystem is expected to pose a significant challenge.

## 3. Impact of Broodstock Nutrition on Reproduction

Broodstock nutrition represents the main factor affecting reproductive performance in farmed fish, with further consequences on the development and survival of embryos and larvae [80,81]. During oocyte maturation, maternal reserves, built up through proper nutrition, are invested in the yolk sac formation that, in turn, will constitute the first energy and nutrient substrates for the developing embryo [81]. Consequently, the early nutritional environment of fish larvae (from fertilization to yolk absorption) is fixed before fertilization and will sustain the newly hatched fish until the occurrence of exogenous feeding [82]. For that reason, diet formulation and feeding frequency must be considered as a crucial point in farmed broodstock nutrition. In this regard, particular emphasis must be given to the essential fatty acids fraction of aquafeeds, especially polyunsaturated fatty acids (PUFAs), being essential to sustain highly energy-demanding processes like reproduction, proper gamete maturation, and correct larval development [83,84]. In fact, long-chain PUFAs, such as eicosapentaenoic acid (20:5n-3; EPA), docosahexaenoic acid (22:6n-3; DHA), and arachidonic acid (20:4n-6; ARA) are able to positively modulate the (i) gamete quality of male fish, including sperm velocity and motility; (ii) reproductive processes of female fish, including oocyte maturation, ovulation, fertility, and spawning rate; and (iii) offspring development, including hatching success and larval quality [85,86,87].

### 3.1. Plant-Derived Ingredients

Traditionally, the main source of long-chain PUFAs in dietary formulations intended for farmed fish is constituted by marine-derived ingredients like fish meal (FM) and, particularly, fish oil (FO) [83,88]. These raw materials represent the gold standard in the aquafeed industry but, in the last decades, their use has been severely restricted due to economic and environmental concerns [89]. In this context, the partial or total replacement of marine-derived ingredients in aquafeeds with vegetable meal (VM) and/or oil (VO) has gained increasing attention, mainly due to their higher abundance and lower price compared to FM and FO [90,91]. Particularly, VOs have been reported to be good candidates since they are characterized by increased sustainable production and price stability [92,93]. However, VO sources are often scarce in long-chain PUFAs (mainly EPA and DHA), despite showing a significant amount of shorter-chain precursors like *α*-linolenic acid (18:3n-3; ALA) and linoleic acid (18:2n-6; LA). Despite the fact that FM and FO inclusion rates in aquafeeds have shown a clear downward trend over the last decades [94], their total replacement by plant sources is still not optimal, with potential consequences on fish reproductive performances.

In this regard, due to the suboptimal long-chain PUFA profile of plant-derived ingredients, several studies have been conducted on zebrafish, as a widely recognized model for aquaculture nutrition [95], and on both freshwater and marine fish of commercial interest to investigate how the reproductive performance can be affected by replacing marine-derived ingredients with plant-derived ones. In this context, promising results were obtained in rainbow trout (*Oncorhynchus mykiss*) fed diets in which FO was totally replaced by linseed oil (LO) or sesame oil (SO), over a short-term period [96]. Particularly in rainbow trout males, semen volume, pH, sperm motility, sperm density, and kinematic parameters were not affected after a 7-month feeding trial, leading to the production of functional gametes able to guarantee proper fertilization and consequent eyeing and hatching rates of embryos [96]. Similarly, no differences in egg indices, fertilization, embryos’ survival, and hatching rates were evident in rainbow trout fed diets characterized by the total replacement of dietary FO with LO or SO for about 5 months prior to spawning [97]. In addition, the contents of ARA, EPA, and DHA of eggs from rainbow trout females fed VO-based diets were greater than the dietary concentrations, highlighting the efficient ALA and LA bioconversion to long-chain PUFA in this species to alleviate the dietary deficiencies [97]. The reproductive performance and fatty acid dynamics of embryos of rainbow trout were also evaluated in response to FO’s replacement with a mixture of VOs (LO, canola, olive, sunflower, corn, and coconut oils) in plant protein-rich broodstock diets, during a 3-month feeding trial prior to spawning [98]. Particularly, the use of an FO:VO ratio of 25:75 led to positive results in terms of fertilization rate, survival at eyed-eggs stage, eggs weight and diameter, and hatching rate; however, the complete replacement of FO with VO blends resulted in a pronounced reduction in the long-chain PUFA content of eggs which may have consequently affected their quality parameters [98]. Longer-term studies (3 years) revealed that rainbow trout reared entirely on plant-based diets, totally deprived of marine raw materials, can successfully produce eggs in which neo-synthesized n-3 long-chain PUFAs are accumulated, leading to viable offspring, despite a reduction in egg size [99]. In addition, complete FM replacement with cottonseed over a 3-year trial did not affect the rainbow trout males’ reproductive performance in terms of steroid hormones, the sperm concentration, and motility but resulted in reduced fertility in females [100].

An additional drawback related to the use of high inclusion levels of vegetable ingredients is the presence of toxic metabolites like zearalenone, a mycotoxin produced by several species of *Fusarium* moulds that are commonly found in plant products used as raw materials for aquafeed production [101]. In this regard, Woźny et al. [102] observed that feed-borne exposure to zearalenone negatively affected gonadal development in rainbow trout, with occurrence of intersex fish and sex-reversed (feminized) males, and offspring survival. These results bring attention to the potential contamination of plant-derived ingredients that can be detrimental to different phases of the reproductive cycle, from gonad maturation to reproductive events.

### 3.2. Nutritional Programming

Freshwater fish are generally able to elongate shorter-chain precursors to long-chain PUFAs, even if supplying them preformed is more advantageous due to their pivotal role in several physiological processes, including reproduction [103]. Conversely, marine fish have had no evolutionary pressure to retain the ability to endogenously produce lc-PUFAs and thus they must be provided through the diet [104]. In this context, an interesting strategy to optimize the use of plant-derived ingredients in marine fish is represented by the production of specimens characterized by a higher capacity of long-chain PUFAs.

Environmental factors in the periconceptional and early developmental stages, including nutrition, can deeply influence the offspring metabolic pathways to prepare them for nutritional challenges through the nutritional programming [105]. In this context, research has been focused on (i) challenge nutrition during very early life history that can improve the ability of fish to effectively rely on suboptimal diets later in life [106,107,108,109,110] and (ii) the maternal role in progeny programming, producing offspring better adapted to being fed suboptimal diets and expanding the existent knowledge of how broodstock nutrition can deeply influence the fish’s reproductive performance, as well as the embryo and larval development.

The effect of a broodstock diet on nutritionally programmed progeny has been widely explored in gilthead seabream (*Sparus aurata*), in which the optimal development of eggs and, then, embryos relies on a proper provision of sufficient quantities of essential nutrients, particularly long-chain PUFA, due to its scarce bioconversion ability [111]. Gilthead seabream broodstock challenged with a 60% replacement of FO by LO resulted in offspring with a greater ability to cope with low FM and FO diets and this nutritional programming persisted with a modulation of selected genes in the offspring, which regulated energy metabolism in the liver, even after 16 months, when fish reached the reproductive age [112,113]. However, higher LO levels (80% of FO replacement) markedly reduced spawning quality, larval survival, and larval and juvenile growth [112,113] as a consequence of the deleterious effects of a too drastic n-3 long-chain PUFA reduction in broodstock diets [114]. Similarly, Xu et al. [115] highlighted that, in gilthead seabream broodstock, the provision of a diet in which FM (15%) was replaced by VM, despite not altering the fish’s reproductive performance, negatively affected the offsprings growth and their ability to use 18C-PUFA through a downregulation of the fatty acyl desaturase 2 (*fads2*). Conversely, the same FM-VM ratio combined with an FO replacement with a mixture of VO (LO, palm oil, and rapeseed oil) in broodstock diets resulted in a reduced female fecundity and egg EPA and DHA contents. However, an upregulation of hepatic *fads2* and elongase of long-chain PUFAs (*elovl6*) was detected in juvenile progenies, highlighting an enhanced biosynthesis of these fatty acids in the offspring related to the FO replacement with VO [115].

In light of these results, optimizing broodstock diets to obtain a nutritionally programmed progeny without compromising spawning quality represents a crucial point. In this regard, a recent study proposed to challenge gilthead seabream broodstock for three months during the spawning season with a diet characterized by mixture of 20% FO and 80% rapeseed oil, after being fed for three months before the spawning season with a low FM (5%) and FO (3%) diet, high in LA and ALA and low in long-chain PUFAs to induce a *fads2* upregulation [116]. Interestingly, broodstock with high blood levels of fads2 showed better reproductive performance, in terms of fecundity and sperm and egg quality. This study consequently identified a feasible percentage reduction in long-chain PUFAs (ARA, EPA and DHA reduced to 0.4, 6.6 and 8.4% of total fatty acids) for a diet designed to induce nutritional programming in the offspring that, on the other hand, preserved reproductive performance in broodstock [116].

### 3.3. Alternative Ingredients

The aquaculture sector has experienced the introduction of functional ingredients that are able to replace unsustainable marine-derived raw materials or fill the gaps originated from the introduction of suboptimal plant-derived ingredients, even at low concentrations, in fish diets [117]. These solutions can represent an alternative dietary-derived solution to positively modulate the overall welfare and reproductive performance of fish fed diets with reduced levels of marine-derived ingredients. Particularly, potential solutions of both animal and microbial origin have been tested in order to evaluate the effects of alternative ingredients on fish reproduction.

Considering the ingredients of animal origin, the use of crustacean biomass as a suitable and cost-effective choice to improve the fish’s reproductive performance was tested [118]. Particularly, it has been evidenced that supplementing *Artemia salina* biomass (2.5%) or *Palaemon elegans* (7.5%) in a plant-based diets low in FM (17%) for European seabass (*Dicentrarchus labrax*) broodstock resulted in improved egg quality (egg diameter and oil droplet size), fecundity, viability, fertilization, and hatching percentages, with a positive modulation of hormones involved in reproduction [118]. Furthermore, partial FM replacement with *Rapana venosa* meal up to 50 g/kg in diets for rainbow trout broodstock has been shown to not significantly affect fish reproductive parameters and did not alter the morphological architecture of different target organs, including the intestine, liver, and muscle [119], usually analysed for determining the overall welfare of farmed fish in response to alternative ingredients [120,121].

Dried microbial biomasses from protist, cyanobacteria, and microalgae have gained interest for their content in proteins, lipids (particularly PUFAs), vitamins, and pigments with antioxidant properties and for their consequent beneficial roles on fish welfare, even at low dietary concentrations [122,123]. Considering microalgae, a recent study on zebrafish evidenced that dietary FM replacement with *Chlorella* sp. meal was able to enhance the fish’s reproductive performance [124]. Particularly, the highest inclusions of *Chlorella* sp. meal tested (40 and 50 g/kg) ensured the highest egg production, hatching rate, and larval survival at 6 days post-fertilization (dpf) [124]. The cyanobacterium spirulina (*Arthrospira platensis*) has gained notable attention for aquafeed formulation due to its antioxidant properties and the abundance of ALA and LA, which are precursors of arachidonic acid, fundamental for prostaglandin synthesis involved in the modulation of steroidogenesis, oocyte maturation, and ovulation [125,126,127]. However, the positive effect of spirulina on fish reproduction has been widely explored only in ornamental fish, including the three-spot gourami (*Trichopodus trichopterus*) [128] and the yellowtail cichlid (*Pseudotropheus acei*) [129]. Considering species of commercial interest, Lu and Takeuchi [130] investigated the reproductive performance of Nile tilapia (*Oreochromis niloticus*) fed solely on raw spirulina, concluding that this dietary solution was able to maintain normal reproduction throughout three generations.

Finally, in light of their fatty acids profile, microbial biomass from certain protists species has gained attention in the aquafeed production sector [131]. In this regard, thraustochytrids are protists that have lost photosynthesis, and they are able to store large amounts of long-chain PUFAs in heterotrophic conditions [132]. Particularly, *Schizochytrium* sp. is characterized by a high lipid content (55–75% in dry matter) and up to 49% DHA of total lipids [133]. Interestingly, the inclusion of *Schizochytrium* sp. oil (6.9%) in a plant-based diet intended for rainbow trout broodstock resulted in reproductive performance and egg quality comparable to those observed in fish fed a conventional commercial FM- and FO-rich feed and led to a significant increase in fry survival [134]. Additionally, the 4-month-old offspring obtained by the broodstock fed the plant-based diet enriched with *Schizochytrium* sp. oil were characterized by a notable growth improvement and a positive modulation of long-chain PUFA biosynthesis and β-oxidation pathways when challenged with the same diet over a 1-month feeding trial [134]. Interestingly, the *Schizochytrium* sp. was also proposed as a solution to improve the nutritional profile of black soldier fly (*Hermetia illucens*) prepupae meal (HM), subsequently used as feed ingredients in diets for zebrafish (*Danio rerio*) [135,136].

The full-fat HM represents a widely explored raw material for diet formulations in aquaculture, characterized by a proper protein content but also by an unbalanced fatty acid profile, high in saturated fatty acid (SFA) and low in long-chain PUFA [137]. This fatty acid profile was previously addressed as the main factor affecting the reproductive performance of female zebrafish fed, over their whole life cycle, 50% of an FM replacement with not-enriched full-fat HM, resulting in a reduction in spawning rate and in an increased number of previtellogenic and atretic oocytes [138]. However, in that case, no alteration in the hatching rate was evident, highlighting the effort that female fish put in the reproductive event, choosing quality rather than quantity of spawned eggs [138]. Interestingly, using *Schizochytrium*-enriched HM, 50% inclusion with respect to FM did not impair zebrafish female reproduction, ensuring performances similar to those observed in fish fed a commercial FM- and FO-based diet; an adverse effect on spawning, hatching, and oocyte classes’ frequency was only evident at 75% or total FM replacement [136]. Furthermore, the long-term provision of the HM-based diets (characterized by 25, 50, 75, and 100% of FM replacement with *Schizochytrium*-enriched HM) during the whole life cycle of zebrafish (from larvae to adult) resulted in offspring better adapted to cope with low long-chain PUFA dietary formulations that did not show adverse effects on their growth and welfare [135].

In general, the main drawbacks regarding microbial biomasses as ingredients for fish diets are represented by the high production costs that still limit their use in the aquaculture sector [139,140] and by the presence, in certain species, of a thick cell wall that can impair nutrient absorption, especially in carnivorous fish [141]. However, the use of a combination of different microalgal species may represent a solution to enable higher dietary inclusion levels respect to those achievable using a single species [142].

### 3.4. Functional Feed Additives

Several studies have been conducted to improve the broodstock diet quality by enrichment with functional additives. Al-Feky et al. [143] evaluated the effects of different supplementation levels of taurine (0, 5, 10, and 15 g/kg) on the reproductive performance of Nile tilapia broodstock fed soybean meal-based diets. Spawning performances, including spawning frequencies, total number of spawning per tank, number of spawning per female, and absolute fecundity, were all significantly improved with increasing levels of dietary taurine up to 10 g/kg. Furthermore, the eggs produced from broodstock fed 10 g/kg taurine exhibited significantly higher hatchability and required a shorter time for hatching and for yolk sac absorption. Higher larval weight was also evident in response to this taurine dietary level [143].

The supplementation of vitamins, which cannot be synthesized by fish, may result in positive effects on gamete quality [144]. Vitamin C (ascorbic acid) and vitamin D have been the most used vitamins for improving broodstock diets, and positive effects on sperm motility have been reported in several fish species, including Nile tilapia [145], African catfish (*Clarias gariepinus*) [146], and Senegalese sole (*Solea senegalensis*) [86]. In addition, vitamin C has been shown to induce positive effects on sperm features in lebranche mullet (*Mugil liza*), in which dietary supplementation between 107 and 216 mg/kg optimizes the spermatic quality in this fish species, improving motility time and rate as well as spermatic density [147].

Finally, the dietary supplementation with probiotics, especially probiotic bacterial strains, led to a significant improvement in reproductive performance in farmed fish [148]. Particularly, the provision of 4 × 10^9^ CFU/kg diet of probiotic Bio-Aqua^®^ twice a day for 8 weeks before spawning season in rainbow trout resulted in an improvement in egg diameter, relative fecundities, fertilization and hatching rates, and eyed-egg and alevin survival rates, as well as in an anticipation of eyeing, hatching, and yolk sac absorbing stages [149]. Due to an increase in egg production and egg size in probiotic-fed treatments, this feed additive may represent a suitable ingredient to increase high-quality egg production in rainbow trout breeders [149].

### 3.5. Feed Restriction Practices

Given the importance of well-formulated diets for broodstock fish that ensure proper reproductive performance, it should be considered that the largest cost for modern aquaculture is represented by aquafeeds, especially those formulated for broodstock (representing between 60 and 80% of production cost), demonstrating the need for research focused on feed provision management [150]. In this regard, practices like feed restriction are of economic interest for fish farmers. However, restriction regimens superior or equal to half food ration can severely affect spawning success [151], as well as egg size and quality [152], and can lead to an arrest of reproductive development [153]. Interestingly, Cardona et al. [154] demonstrated that a moderate, yet significant, feeding restriction of 20% in rainbow trout, applied during the last five months before the second reproduction, did not affect egg production and egg quality, providing the first evidence of the acceptable level of feed restriction. This can represent a compromise between reducing the feed-associated costs and the maintenance of proper broodstock trout reproductive performance.

## 4. The Use of Model Species to Investigate Reproductive Challenges in Teleosts

Aquatic species, including teleosts, are especially susceptible to the toxic effects of pollutants due to their constant exposure to low concentrations of contaminants that continually enter the aquatic environment. In this regard, numerous studies have been conducted to investigate the reproductive toxicity of anthropogenic xenobiotics widely present in the environment, mainly using teleost model species, primarily *Danio rerio* for freshwater fish and, to a lesser extent, the Japanese medaka (*Oryzias melastigma* and *Oryzias latipes*) for saltwater fish. Model species, which are often chosen for their biological relevance, ease of maintenance in laboratory settings, and well-documented genetic and physiological profiles, provide valuable insights into the mechanisms by which pollutants impact fish health and ecosystems [155]. These species allow us to extrapolate findings to other, often more complex, wild and farmed fish species [95]. By examining the responses of model organisms to various contaminants, it is possible to identify potential risks, understand modes of action, and predict ecological consequences. This research is crucial for informing regulatory frameworks, improving environmental monitoring, and developing mitigation strategies to protect both wild fish populations and aquaculture operations from the adverse effects of pollution. Among these contaminants, Endocrine Disruptive Chemicals (EDCs) are those with the most detrimental effects on reproduction, due to their capacity to interfere with the hypothalamus–pituitary–gonad (HPG) axis by affecting endogenous hormone synthesis, secretion, transport, binding, or elimination [156]. The most prevalent EDCs in the environment are classified as plasticizers, primarily represented by bisphenols (i.e., BPA, BPS, BPF, BPC, BPB, and BPAF) and phthalates (i.e., DEHP, DiNP, DBP, DiDP, and DPHP). Per- and polyfluoroalkyl substances (PFASs) are another significant category of EDCs that are almost ubiquitous in the environment, with the main compounds being Perfluorooctanoic acid (PFOA) and Perfluorooctanesulfonic acid (PFOS). A vast body of the literature exists concerning the reproductive toxicity exerted by these pollutants on gametogenesis progression, gamete quality, and reproductive efficiency. Over the years, the findings have been summarized in numerous reviews [157,158,159,160,161,162,163].

In addition to plasticizers and PFAS, various other environmental contaminants can act as EDCs, leading to reproductive toxicity due to their widespread presence in the environment. Among these contaminants, heavy metals constitute a broad class of metallic elements characterized by high density and atomic weights, such as copper (Cu), mercury (Mg), cadmium (Cd), arsenic (Ar), zinc (Zn), iron (Fe), and chromium (Cr). These metals are extensively utilized across various industries due to their unique properties, finding applications in manufacturing, construction, electronics, and even healthcare [164]. In recent years, widespread industrialization, urbanization, and agricultural practices have resulted in increased heavy metal pollution across various environmental compartments, including soil, water, and air. Once released into the environment, heavy metals can endure for extended periods, causing chronic exposure and having adverse effects on various physiological processes, including reproduction.

Furthermore, the inadvertent release of pharmaceutical compounds into aquatic environments is also a source of concern due to their possible toxic effects on aquatic wildlife’s reproductive activities. These chemicals include a wide range of medications, comprising antibiotics and antidepressants, and are able to enter the waterways through various routes, such as wastewater discharge, improper disposal, and excretion by humans and animals [165]. Fish can ultimately be exposed to pharmaceutical compounds directly from the water or through the food web, also leading to the bioaccumulation of these chemicals in their tissues [166,167]. Moreover, another cause of pollution in the aquatic ecosystem is represented nowadays by the intensive use of pesticides because of the widespread use of this class of compounds, primarily in agriculture, to control pests or unwanted organisms, such as invasive weeds, insects, and fungi. This results in their entry into waterways through runoff from fields, accidental spills, or improper disposal [168].

This section has focused on summarizing the information obtained regarding the reproductive toxicity resulting from exposure to heavy metals, commercial pharmaceuticals, and pesticides in two widely used teleost models, the zebrafish and the Japanese medaka. The reported effects of these compounds on reproductive success aim to illuminate their molecular mechanisms of action and potential implications for future generations.

### 4.1. Heavy Metals

Among heavy metals, copper, zinc, cadmium, mercury, iron, and chromium are the most used in different manufacturing activities, resulting in a wide presence in the environment. For this reason, many studies have evaluated their impact on fish reproduction.

Considering Cu, the findings highlight the endocrine disruptive ability of this xenobiotic by altering fish steroidogenesis and gonadal architecture in both females and males, despite not showing any effect on fertility and fecundity. In the form of Copper (II) sulfate (CuSO_4_), this heavy metal finds applications in agriculture as a fungicide and fertilizer. Therefore, its runoff from agricultural fields could lead to its entrance in aquatic environments, posing a severe risk for teleost health. Recently, the effects of CuSO_4_ on zebrafish reproduction were investigated, finding that 40 μg/L, a dose in the range of environmental concentrations, was able to alter the expression of master genes involved in reproduction along the HPG axis during chronic exposure (30 days) [169]. The alteration of genes involved in the HPG axis and the steroidogenesis finally resulted in a negative impact on the gonadal architecture, as shown by the decreased number of mature and secondary follicles in females and the number of spermatozoa in males. These results were also supported by the analysis of gonadosomatic index (GSI) which decreased in both sexes [169], suggesting the strong reproductive toxicity of CuSO_4_. Similarly to CuSO_4_, chronic exposure (20 days) to 50 and 100 μg/L Cu oxide nanoparticles, applied to industrial chemical catalysis [170], was observed to alter the mRNA levels of genes belonging to the HPG axis and encoding steroidogenic enzyme and hormone receptors in a sex-specific fashion, thus resulting in alterations in steroid hormone levels without exerting a sex-specific effect and, therefore, in both testicular and ovarian architecture alterations [171]. Despite the clear reproductive toxicity evidenced by both CuSO_4_ and Cu oxide nanoparticles, their impact on the reproductive capacity of the fish, in terms of fertility and fecundity, was not explored. In this regard, to the best of our knowledge, only one study conducted in 2007 aimed to investigate if Cu food administration could affect zebrafish reproduction. Exposure to 100, 500, or 1000 μg Cu per g food in the form of CuSO_4_ for 220 days did not change either the number of eggs spawned or fertilization of F0. Likewise, the F1 embryo’s malformation up to 36 h post fertilization was not affected by Cu parental exposure [172].

Other than Cu, chemicals containing Zn are able to affect reproduction mainly by the induction of oxidative stress at the gonadal level, finally resulting in increased apoptosis levels. Considering this, zinc pyrithione (ZPT), with applications in the industry as algaecides and fungicides, has been used for this purpose in medical contexts, paint formulations, and textile manufacturing. Male zebrafish exposed to 0.15 and 0.30 µM concentrations of ZPT showed reduced activity of CAT, T-SOD, and GSH-PX and an increase in Malondialdehyde [173]. Concomitantly, TUNEL-positive cells and caspase 3 activity were increased. Collectively, these alterations led to a dose-dependent reduction in sperm [173]. This toxicity was also confirmed by the identification of 409 DEGs associated with testicular injury through RNA-seq and the analysis of steroid hormone levels also suggested the endocrine disruptive capacity of ZPT [173]. In the Japanese medaka, the exposure to 1, 5, and 10 μg/mL of zinc oxide nanoparticles (nZnOs) and 1, 5, 10, and 20 μg/mL ZnSO_4_ for 1 week affected both reproductive behavior and steroidogenesis [174]. On the other hand, in males, nZnO was able to induce sperm ROS, causing sperm quality alterations, while in females the nZnO-induced ROS increase led to follicular growth arrest and atresia increase, finally leading to a subsequent fertility decrease caused by this contaminant in both sexes [174]. Finally, in order to evaluate the effects of Zn on sex differentiation and development, exposure of zebrafish to 650 μg/L of Zn during the first five days post fertilization (dpf) resulted in an increase in female to male ratio and hatching delay in the F0 and, interestingly, also in the progeny F1 [175]. Considering this, Zn toxicity could last after recovery from the exposure and could also affect the next generations.

Cd is another heavy metal that is present in a wide range of environments. Exposure to Cd and Cd-containing molecules was demonstrated to affect fertility and embryo development. The toxicity of Cd on female reproduction was evaluated in *Danio rerio* by exposing the specimens to 10 μg/L Cd continuously, 20 μg/L Cd for 1 day every 2 days, or 30 μg/L Cd for 1 day every 3 days for a total of 48 days [176]. Exposure to the first two concentrations was able to decrease the percentage of spawned eggs, possibly as a consequence of the decreased plasma levels of VTG and E2 and the perturbation of mRNA levels of brain–liver–gonadal axis genes. A reduction in GSI and hatching rate, coupled with increased embryo mortality, was instead evident in all the Cd concentrations tested [176]. Chronic exposure (30 days) to 1 μmol/L of cadmium chloride (CdCl_2_), an industry dye, also disrupted zebrafish female reproduction [177] by reducing the number of mature oocytes and causing the induction of developmental malformations in the offspring [177]. Moreover, deep-sequencing analysis shed light on the pathways underlying this reproductive toxicity, mainly affecting the estrogen metabolism, meiosis, and vitellogenin synthesis of oocytes [177]. In addition to the reproductive toxicity of Cd on female zebrafish, another study demonstrated that maternal exposure to 8.9, 17.8, and 35.6 µM of Cd^2+^ affected the pair-spawning success rate at all the concentrations tested, with the highest one also reducing the hatching percentage at 36 hpf [178]. Considering the effects of Cd on male fish reproduction, the exposure of zebrafish spermatozoa to Cd in vitro (0.5, 5, and 10 μg/L of Cd), demonstrated the direct effect of this pollutant on sperm motility as well as plasma membrane and DNA integrity [179]. Despite the interesting results found by these authors, no test was conducted on the capacity of the treated spermatozoa to fecundate the oocyte, letting us only hypothesize the possible implications of Cd exposure on the zebrafish fertility rate.

The presence of Hg in the aquatic environment represents another source of severe concern. For this reason, the impact of this heavy metal on female and male reproduction was evaluated in zebrafish, finding its ability to alter reproduction by increasing ROS levels and interfering with the HPG axis, also leading to detrimental effects on progeny. Chronic exposure (30 days) of zebrafish adults to 15 and 30 μg/L mercuric chloride (HgCl_2_) increased the expression of genes encoding the antioxidant enzymes in both the ovaries and testes and higher MDA and GSH testicular levels. These results, together with changes in the HPG axis and male steroidogenesis, were correlated with the presence of atretic oocytes in females and a decrease in the number of spermatozoa in males [180]. The effects of zebrafish embryonic exposure to 0.6, 3, or 15 μg/L Hg^2+^ until 5 dpf caused a detrimental effect on the expression of genes related to hormone and neuropeptides synthesis, leading to reproductive behavior alteration and subsequent consequences on spawning and fertilization rate [181]. Interestingly, the aforementioned effects on reproduction and reproductive behavior were not evident in the untreated F1 generation [181], indicating that the toxic effects were not transmitted to the progeny. Hg^2+^ exposure decreased zebrafish fecundity and delayed gonadal development, resulting in the GSI decrease in F0 [182], a condition that persisted, to a lesser extent, in the F1 females and was absent in F1 males and in both sexes at F2 generation. Although these results may not represent a risk for future generations, the inter- and transgenerational effects of mercury could be influenced by the form in which this compound is applied and by the window of exposure. In this regard, 24 h early-life exposure to methylmercury (MeHg) at different concentrations (1, 3, 10, 30, and 100 nM) was observed to trigger sperm epimutations related to neurotoxicity at 30 nM in the F2 generation, even though the F0 was not affected [183].

Japanese medaka was used as model to determine the effects of two other heavy metals, Fe and Cr. Considering Fe, the effects of 21-day exposure to 5 and 20 mg/L of Fe^2+^ or iron oxide nanoparticles (nFe_3_O_4_) were assessed on *Oryzias melastigma* reproduction, finding that 20 mg/L of nFe_3_O_4_ decreased the fecundity and increased the occurrence of abnormal immature oocytes inside the ovaries, a mechanism that could be due to the contaminant inhibition of antioxidant SOD and GPX activities both in the ovaries and the brains of the specimens exposed [184]. Regarding Cr, the reproductive toxicity induced by 4 mg/L of Cr (VI) (administered as K_2_Cr_2_O_7_) was investigated in Japanese medaka exposed for 3 months [185]. The results evidenced a decrease in male GSI, whereas in females, a reduction in spawned eggs and fertilization rate was observed [185], correlating well with an increased GSH/GSSG ratio in the ovaries and a reduction in both CAT and SOD levels in the testes [185].

### 4.2. Pharmaceuticals Compounds

A wide range of medications can enter the aquatic environment after human consumption and, among them, psychotropic molecules, such as antiepileptics, antidepressants, and antipsychotics are widely represented because of their chemical properties, which make their removal through wastewater treatment plants difficult. As a consequence, their presence is commonly found in different fish tissues and they were observed to be able to cause reproductive and developmental toxicity in zebrafish, as recently reviewed [186]. To the best of our knowledge, till date, only one study evaluated the reproductive alteration induced by psychoactive drugs on *Oryzias melastigma*. Fluoxetine, a selective serotonin reuptake inhibitor widely prescribed as an antidepressant, is highly environmentally present because of human consumption. Chronic exposure to environmentally relevant concentrations of Fluoxetine (0.1, 0.5, 1, and 5 µg/L) did not alter either egg production, fertilization, and the spawning rate or the hatchability of fertilized eggs, despite increased occurrence of developmental abnormalities [187]. Although parental GSI, liver vitellogenin content, and gonadal steroidogenesis were again unaffected, an effect on steroidogenesis was evidenced by the increase in E2 plasma levels by the lowest concentration, highlighting the disruptive chemical ability of this drug [187].

Analgesics represent another class of drugs abundantly found in water due to the anthropogenic action. Chronic exposure (28 days) to acetaminophen (ACE) at environmentally relevant concentrations (0.5, 5, and 50 µg/L) in zebrafish altered gonadal steroidogenesis in both sexes, increased vitellogenic follicles’ dimension and number, and as follicular atresia in females, while in males, ACE triggered testis cellular apoptosis [188]. The reproductive toxicity of another anti-inflammatory drug, Ibuprofen, was evaluated by chronically exposing (36 days) zebrafish to 0.5, 2.4, 11.5, and 55.2 µg/L, finding no effects on F0 fertility and fecundity, as well as F1 generation hatching success [189]. On the other hand, Ibuprofen exposure (1, 10, and 100 µg/L) for 6 weeks was able to increase the number of eggs of *Oryzias melastigma* per reproductive event, despite decreasing the number of spawning events per week [190]. Finally, Diclofenac, another drug highly prescribed for its anti-inflammatory activity, led to a reduction in fertility and fecundity in medaka fish exposed to 37 and 78 μg/L for 14 days [191]. This effect was gradually rescued by maintaining the fish under standard conditions for other 14 days, suggesting that the effects exerted on reproduction could be reversible to a certain extent. Interestingly, another study showed that even though parental fertility was unaffected by chronic exposure (3 months) to 10 mg/L of Diclofenac, this contaminant still led to a complete impairment of F1 medaka embryo hatchability [192], indicating the transmission of this anti-inflammatory toxicity to the progeny.

Antibiotics and antibacterial compounds are widely, and sometimes inappropriately, prescribed and used nowadays to fight human and animal infections. Their extensive use over the years has led to the accumulation of this class of compounds in the environment, resulting in toxicity at different levels to non-target organisms. Over the years, evidence accumulates regarding their ability to impair fish reproduction by acting as EDCs and inducing oxidative stress. In this regard, long-term exposure (from 2 hpf to 120 dpf) to environmentally relevant concentrations (1 and 5 µg/L) of Sulfamethoxazole (SMZ), an antibiotic of clinical and animal husbandry, was observed to act on the G protein-coupled receptor 54, which in turn stimulates the synthesis and release of GnRH in the brain. This outcome led to an increase in FSH, LH, and E2 gonadal levels, thus resulting in an increase in adult mature oocyte proportions [193]. SMZ and norfloxacin (NOR), a fluoroquinolone, both singularly or in combination (2, 20, and 200 μg/L) were able to diminish zebrafish’s daily egg production and increase embryo mortality and malformations at the highest dose [194]. Another antibiotic, Ciprofloxacin (CIP), was observed to act as an endocrine disruptor in zebrafish males exposed to 15.6 µg/mL for 21 days by affecting male steroidogenesis and altering the expression of testis gonadotropin receptors, genes related to the endocrine system, signal transduction, and membrane transport [195]. Moreover, zebrafish’s exposure to 6.25, 12.5, and 25 mg/L to β-diketone antibiotics (DKAs) from 6 hpf to 144 hpf [196] showed a disruption in steroidogenesis in a dose-dependent fashion once these embryos reached adulthood and triggered gonadal ultrastructural damage in both sexes, indicated by TEM microscopy [196]. A reduction in egg production, increase in unfertilized eggs, and decrease in hatchability was also evident and, collectively, these alterations also led to transcriptome alteration in F1, highlighting that parental exposure could also result in detrimental effects for future generations [196]. The antibacterial and antifungal chemical Triclosan (TCS) is widely present in the environment due to its extensive use in personal care products. Its exposure at environmentally relevant concentrations (0.4, 4, or 40 μg/L) reduced the fertility and fecundity of F0 zebrafish, an effect that was no longer observed in the F1 generation [197]. Recently, in another study, zebrafish’s exposure to TCS at 2, 20, and 200 μg/L for 150 days demonstrated that the highest dose reduced the male’s fertilization capacity, while all the concentrations reduced embryo hatchability when mating treated males with untreated females [198]. Noteworthy, both female and male specimens also presented a sex-specific alteration of steroidogenesis and VTG whole-body levels, which could be link to the alteration of the testicular structure disruption and the reduction in mature spermatozoa in males and to the increased number of immature oocytes in female [198]. Finally, the long-term (120 days) effects of Cefadroxil (1, 7.8, 84.8, 718.9, and 8883.1 µg/L) and Cefradine (1, 7.1, 73.9, 724.6, and 7758.5 µg/L) on Japanese medaka reproduction were evaluated, showing that both contaminants decreased the number of eggs produced per reproductive pair, also evidencing their ability to affect steroidogenesis in a sex-specific fashion [199].

Nowadays, the link that interconnects the gut microbiota and reproduction is well known, highlighting that intestinal dysbiosis is able to negatively affect reproduction [200,201,202,203]. The results obtained regarding antibacterial and antibiotics exposure to zebrafish and medaka generally agree on the negative effects that these chemicals pose on reproduction. Despite this, since these chemicals could deeply affect gut microbiota composition, no evidence has been reported until now on the possible effects that these compounds could exert at the intestinal level and, consequently, at the reproductive level. Further investigations are needed to fill this gap of knowledge, building a comprehensive picture regarding this issue.

### 4.3. Pesticides

During the last decade, the effects of different classes of pesticides have been evaluated on the reproduction activity and early development of *Danio rerio*, demonstrating their negative effects on reproductive physiology at environmentally relevant concentrations. The results providing information about the pesticides’ implications on reproduction and development in zebrafish, including those obtained through omics approaches, were recently reviewed [163], whereas those obtained using Japanese medaka as an experimental model will be summarized in this section.

Atrazine is a well-known herbicide widely present in the environment due to its massive applications in agriculture, with negative reproductive effects demonstrated on teleosts. In the Japanese medaka, atrazine (0.5, 5, and 50 µg/L) exposure for 38 days led to an alteration in steroidogenesis, altering the ovulation capacity and decreasing the egg production [204]. The same experimental design was also applied in a subsequent study to further investigate the mechanism underlying the reduced egg production, finding a dose-dependent decrease in the expression of genes involved in vitellogenesis and zona pellucida glycoprotein in the eggs, also affecting steroidogenesis and the HPG axis [205]. On the contrary, other studies, both using similar atrazine concentrations and exposure windows (0.6, 5.5, and 53 µg/L for 35 days) of the aforementioned study, or modifying the exposure regime (9.4, 48, 74, 97, and 244 μg/L for 28 days), found no reproductive effects of this herbicide on Japanese medaka reproduction [206,207]. Interestingly, even though the exposure to atrazine at 5 and 50 μg/L during the first 12 days of development (an important time window for epigenetic reprogramming of primordial germ cells and sex determination) did not affected F0 sperm quality, it reduced that of the F2 generation, thus leading to a reduction in the fertilization rate of the males exposed to the lowest concentration [208]. At the molecular level, the observed reproductive toxicity could be explained by the changes in DNA methylation and the expression of genes involved in steroidogenesis in the F2 generation [208], suggesting that even though atrazine reproductive effects are not evident in the parental generation, epigenetic modifications could be transmitted, leading to a reproductive toxicity of non-exposed generations.

The effects on medaka reproduction of another herbicide, Diuron (5, 50, 500, and 5000 ng/L) from 0 dpf to 180 dpf were evaluated, evidencing its detrimental effect on male reproduction by affecting HPG axis-related gene expression, thus altering gonadal architecture and reducing the fecundity and also leading to growth reduction in the F1 generation [209]. In this regard, another study from the same authors investigates the intergenerational effects of this contaminant (500, and 5000 ng/L), showing that Diuron affected F1 generation hatchability and delayed embryo development. Moreover, after reaching sex maturity, these individuals showed alterations in steroidogenesis, *VTG* transcription, and germ cells proportion [210]. This could be due to the increased mRNA levels of DNA methyltransferase along the HPG axis, suggesting the involvement of epigenetic modifications in the transmission of reprotoxic effects to the following generation [210].

Considering the reproductive toxicity of insecticides, Diazinon exposure at 2.9, 5.2, 10.3, 19.8, and 40.2 μg/L to female and male Japanese medaka (at 12-weeks post fertilization) from the F0 to F2 generation led to a dose-dependent reduction in the number of spawned, fertilized, and hatched eggs, also affecting the F1 growing parameters and secondary sexual characters in a dose-dependent manner [211]. These results agree with another study in which 20 μg/L of Diazinon was shown to increase the time to first spawn and reduced the fecundity, leaving the hatch rate of the embryos unchanged [212]. The exposure of female medaka fish to 1, 10, and 100 ng/L Diazinon showed that this contaminant affected the HPG axis in a similar way, with respect to males, and also affected female GSI and the percentage of mature oocytes, while the highest dose did not affect female medaka reproduction [213]. Similarly to males, the exposure of females to this toxicant affected F1 fecundity and viability [213], further confirming that parental exposure can act on the progeny, impairing embryo development.

Moving to anti-fungi chemicals, exposure to the azole fungicide Difenoconazole (1, 10, 100 and 1000 ng/L) for 180 days, starting right before fertilization, led to a negative impact on the transcription of genes involved in the HPG axis and a decreased GSI in males exposed to the highest concentration, while all concentrations led to an increase in spermatogonia and spermatocytes number and a concomitant decrease in the sperm cell number [214]. In the same study, the toxicity of this pollutant on F1 fertilization, hatchability, and sperm swim-up was also evaluated, finding that all these parameters were affected by the contaminant parental exposure [214]. In addition, the exposure to another azole fungicide, Triadimenol, at environmentally relevant concentrations (3–30 μg/L and 300 μg/L), from 0 dpf to 35 dpf (a critical window for gonad development), led to significant modifications of ovary development, such as an increase in previtellogenic oocytes and a decrease in fecundity, also affecting the F1 male to female sex ratio and hatchability [215], and confirming the capacity of azole fungicides to affect the subsequent generations.

## 5. Conclusions

As the Mediterranean Sea continues to undergo transformations, the study of teleost reproduction serves as a critical lens through which we can comprehend the broader implications of human activities on marine ecosystems and work towards their preservation. The challenges of teleost reproduction in the Mediterranean Sea are multifaceted, and addressing these challenges requires a comprehensive understanding of the complex interactions between environmental factors, reproductive biology, and anthropogenic impacts. Studies using model species often focus on specific stressors in isolation, whereas wild teleosts experience a combination of different stressors. Therefore, the cumulative impact of multiple stressors on reproduction may not be fully understood when studying only one or a few factors. In that regard, laboratory settings may not fully replicate the natural environmental conditions that teleosts experience in their habitats, thus providing limited information. However, the results obtained from studies carried out in model species, together with the use of mathematical models, could offer different predictions on energy assimilation and mobilization prior to and during reproduction, which will refine our comprehension of both wild and farmed teleosts. Recently, models that encompass entire life cycles and that quantify reproduction are becoming very useful for farming systems like hatcheries and nurseries, where the early stages of life are crucial.

The use of these new tools, together with an in-depth understanding of the variables that influence the reproduction of organisms, could enable better-informed conservation strategies, sustainable aquaculture practices, and management of wild populations in dynamic environments.
